# Assessment of Dysfunction in the Urinary System as Well as Comfort in the Life of Women during and after Combination Therapy Due to Ovarian and Endometrial Cancer Based on the SWL, II-Q7 and UDI-6 Scales

**DOI:** 10.3390/jcm10061228

**Published:** 2021-03-16

**Authors:** Marcin Opławski, Magdalena Smoczyńska, Beniamin Oskar Grabarek, Dariusz Boroń

**Affiliations:** 1 Department of Gynecology and Obstetrics with Gynecologic Oncology, Ludwik Rydygier Memorial Specialized Hospital, 31-826 Kraków, Poland; bgrabarek7@gmail.com (B.O.G.); dariusz@boron.pl (D.B.); 2 Department of Rehabilitation Psychology, Ludwik Rydygier Collegium Medium, Mikołaj Kopernik’s University CM UMK, 30-835 Bydgoszcz, Poland; msmoczynska2020@gmail.com; 3 Department of Histology, Cytophysiology and Embryology in Zabrze, Faculty of Medicine in Zabrze, University of Technology in Katowice, 41-800 Zabrze, Poland; 4 District Hospital in Chrzanów, 32-500 Chrzanów, Poland; 5 Department of Nursing and Maternity, High School of Strategic Planning in Dąbrowa Górnicza, 41-300 Dąbrowa Górnicza, Poland

**Keywords:** urogynecological examination, brachytherapy, chemotherapy, endometrial and ovarian cancer, quality of life, stress incontinence

## Abstract

This work aimed to assess the influence of oncological combination therapy that was done on endometrial or ovarian cancer and how the urinary system is influenced as well as the quality of life in comparison to a group of female patients after the removal of the uterus with appendages due to endometrial cancer, which did not require the supplementation of therapy after operative treatment. The study included 87 patients with endometrial cancer, where, after the removal of the uterus, there was no need for conducting adjuvant therapy (C), as well as 92 female patients with endometrial cancer or 38 patients with ovarian cancer in whom combination therapy was conducted (group A, B). The assessment of the quality of life was conducted using the questionnaires: Satisfaction Life Scale (SWLS), Incontinence Impact Questionnaire, Short Form (IIQ-7), and Urogenital Distress Inventory (UDI-6) for three, six, nine, and 12 months after the conclusion of oncological treatment. It was observed that there was a statistically significant decrease in the quality of life in female patients who underwent combination therapy in comparison to a group in whose treatment only included surgery (*p* < 0.05). The risk of developing urinary incontinence increases alongside an increase in the scope of the operation and in the case of supplementing treatment with brachytherapy in comparison to chemotherapy.

## 1. Introduction

Tumors within the female sexual system, including breast cancer, constitute approximately 40% of all cancers in women [1]. An increase in the number of cancers mainly involves tumors of the endometrium and ovaries, which is connected with an improvement in the socio-economic situation, including a longer life expectancy, or increasingly common diseases of civilization, such as obesity, arterial hypertension, or diabetes [2,3,4]. Endometrial cancer (cancer of the endometrium) is characterized by the highest rate of disease growth [5]. Endometrial cancer is recognized in the early stages due to the characteristic symptoms in the form of bleeding, whereas ovarian cancer is recognized, due to the lack of symptoms identified, in the late stages of advancements [5,6,7]. In both cases, combination therapy is utilized [7,8,9]. Endometrial and ovarian cancer oth most commonly begin with surgery, in which full radicalism is sought. In the case of endometrial cancer, the adjuvant treatment is brachytherapy and/or radiation therapy, as well as in more advanced cases, chemotherapy, and hormone therapy [8]. Indications for adjuvant brachytherapy and radiation therapy after surgery are still expanding, because they have a positive effect on the remission time of the disease. Combination therapy (surgery plus brachytherapy (VBT) and/or teletherapy (ERBT)—in endometrial cancer or surgery with adjuvant chemotherapy of ovarian cancer) gives the expected results, in the form of remission of the disease, statistically significantly more in the case of endometrial cancer than ovarian cancer [6,7,8,9].

In most cases, combination therapy decreases the quality of life for oncological cancer patients. Disorders arise within the digestive tract, nervous and hematopoietic systems, and additionally affect the mental state of patients [9,10,11]. One of the causes of a decreased comfort of life for these women are ailments that result from disorders in the urinary system, in which stress urinary incontinence (SUI), urge urinary incontinence (UUI), and mixed urinary incontinence (MUI) are all included. Urinary incontinence in women, including SUI, is defined as every uncontrolled leakage of urine, this issue affects between 17% to 60% of women, negatively influencing their quality of life [12,13].

The observations of Strauchon et al. are interesting in the context of the issue of urinary incontinence connected with the oncological treatment of gynecological cancers. The goal of their research was the assessment of the influence of chemotherapy with the use of carboplatin/paclitaxel due to ovarian, fallopian tube, peritoneal, or endometrial cancers in a group of 62 women [14]. Their observations were based on the surveys: social aspects of aging (MESA); the questionnaire IIQ-7, which assesses the influence urinary incontinence has on the everyday functioning of the patient; or, by completing the Urogenital Distress Inventory (UDI-6) form, which assesses the intensification of urinary incontinence symptoms [15]. Based on the comparison of the obtained results before the beginning of treatment, in the middle of the fifth cycle of chemotherapy as well as 6–12 weeks after the end of treatment, they indicated that the treatment method used significantly decreases the quality of life, and it has an influence on the occurrence of urinary incontinence. However, they highlight that further research is necessary [14]. Nonetheless, the research conducted by Ramaseshan et al. is important, who observed that pelvic floor disorders occur statistically significantly more often in patients with cancer of the reproductive organs than in the healthy female population. In the group of women with cervical cancer, the frequency of occurrence for SUI, UUI, and fecal incontinence (FI) was between 24–29%, 8–18%, and 6% respectively. Whereas, after treatment, the aforementioned ailments affected 4–76%, 4–59%, and 0.4–39% respectively. Moreover, in the group of women with endometrial cancer, the differences before and after treatment were the following: 29–36%; 15–25%; 3% vs. 2–44% (UUI, SUI); and, 7–39% (FI), respectively. This highlights that the problem of urinary incontinence amongst oncological patients is significant and socially important [16].

The diagnosis of urinary incontinence requires close cooperation between a gynecologist, urologist, and psychologist. It begins with collecting an interview, gynecological examination, laboratory urine examination, functional tests, and the insertion of a voiding frequency and urine volume card [17]. During diagnosis, a urogynecological examination should be completed, in order to determine the pressure at which involuntary urination occurs when the bladder is filled at the level of 200 mL. Urogynecological examinations are recommended in clinical practice in an early period after gynecological-oncological operations [18].

This study aimed to assess the influence that oncological combination therapy, due to endometrial or ovarian cancer, has on the functioning of the urinary system, on the quality of life when compared to a group of patients after the removal of the uterus with appendages due to endometrial cancer, which did not require the supplementation of therapy after surgery.

## 2. Experimental Section

The study was conducted according to the guidelines of the Declaration of Helsinki and approval by the Institution of the Bioethical Committee operating at the Medical Higher School in Opole, Poland, no. 60/PO/2019 has been obtained for this study. Data confidentiality and patient anonymity were maintained at all times. Patient-identifying information was deleted before the database was analyzed. It is not possible to identify patients on an individual level either in this article or in the database. Informed consent was obtained from all of the subjects involved in the study. The survey was conducted from 01.06.2017 to 01.09.2020, whereas the operations were conducted in the period 01.03.2017–01.09.2019.

Urogynecological examination and survey assessment were collectively done on 217 women, including 87 women forming the control group, 92 women with endometrial cancer undergoing surgical treatment and brachytherapy as well as 38 patients with ovarian cancer in whom, surgery was done and chemotherapy was used, for 100 in each of the three analyzed groups (the examination was performed by one oncological gynecologist who also specialized in urogynecology as well as one clinical psychologist). The control group was formed of 87 women at cancer stage I and grade 1 differentiation (age = 55.11 ± 8.49 years, BMI 28.21 ± 7.91-overweight), in whom a radical surgery involving the removal of the uterus with the vaginal margin and lymphadenectomy due to endometrial cancer was performed. Among these patients, oncological treatment was not supplemented with radiation therapy. The first study group (group A), which was collectively 38 women with diagnosed ovarian cancer, completed the combination therapy and chemotherapy with the desired effect. Within this group of patients, 20 had stage I ovarian cancer (age 55.03 ± 4.11 years; BMI 27.06 ± 4.24-overweight), whereas 18 had stage II ovarian cancer (age 55.66 ± 4.82 years; BMI 28.16 ± 4.55-overweight). In terms of surgical treatment, the removal of the uterus with appendages, appendicitis, networks, small pelvic, and periaortic lymph nodes was carried out. In the examination, only the patients were included, in whom, as a result of surgery, a complete cytoreduction was achieved-a lack of macroscopically visible changes in the peritoneal cavity. Surgical treatment was supplemented with standard chemotherapy (Taxol + Carboplatin). The second study group (group B) also included 92 patients after combination therapy due to endometrial cancer in the IA2-IIIC advancement stage (degree of histopathological differentiation: well-differentiated G1—27 cases, age 68.24 ± 5.07 years, BMI 29.02 ± 5.23—overweight; moderately differentiated G2—65 cases, 67.8 ± 6.19 years, BMI 33.36 ± 10.98—class I obesity). Patients first underwent radical surgery as well as adjuvant treatment using brachytherapy (VBT).

## 3. Urogynecological Examination

Urogynecological examination was only done as a supplementary examination in order to refine the diagnosis. We used VLPP values (Valsalva Leak Point Pressure) in patients where the urogynecological examination was done, which is the lowest, critical intra-abdominal pressure, obtained during the Valsalva maneuver, where leakage occurs. Additionally, we determined that the maximum coil closing pressure was VLPP > 60 cmH20 in order to confirm the SUI.

Urogynecological examination encompasses a complex assessment of the functionality of the bladder and urethra. The examination lasts between 60 to 90 min. It is carried out in an intimate and relatively stress-free environment for the patient. It begins with the measurement of the urine flow measurement, which is based on voiding it by the examined person into a special vessel, while bearing in mind the time that is taken to complete this process, as well as assessing the residual urine after voiding is completed. The urogynecological examination is conducted on a urological-gynecological chair. The patient undresses halfway (lower half of the body). Afterward, the patient lies down on their side with their legs slightly spread. After the area around the mouth of the external urethra is washed with disinfectant, the area is locally anesthetized with a gel that is introduced directly into the urethra. The next stage of the examination is the placement of a catheter in the bladder.

A second catheter is placed in the rectum. Pressure transducers are connected to the catheters. With their help, the change in pressure in the bladder and abdominal cavity is recorded. Prior to testing, the catheters are filled with saline to remove air from them, and the transducers are zeroed to atmospheric pressure. In addition, the electrodes are glued to the anus area of the examined person, thanks to which electromyographic examination of the urethral sphincter muscles is performed.

### Uroflowmetry-Urethra Flow Measurement

Uroflowmetry is performed while using a device called a uroflowmeter. The uroflowmeter measures the volume and mass of the voided urine within a time frame. The result of the uroflowmetry is given in milliliters per second. The uroflowmetry examination should be completed with an evaluation of postvoid residual urine (PVR). The PVR assessment is performed using an ultrasound machine. The uroflowmetry result depends on the age and sex of the patient; hence, the maximum urethral flow in an average young healthy man is above 15 mL/s; this value decreases with age. In healthy men over 60 years of age, results that are above 15 mL/s are considered to be the correct maximum flow (Qmax). In women, the maximum tubular flow is 5–15 mL higher than in men of the same age. The tubular flow rate in ml/s is the result of the contraction force of the bladder detrusor muscle and the resistance of the tubule. The minimum urine volume in the bladder during uroflowmetry should be at least 150 mL. The result of uroflowmetry depends on the strength of the detrusor contraction and tubular resistance.

## 4. Post-Micturition Residual Assessment-PVR

### 4.1. Cystometry

Cystometry is an invasive test, in which the pressures in the bladder and abdominal cavity are measured. Cystometry is performed to assess the urine storage phase of the bladder. This process involves filling the bladder with sterile fluid, which is usually saline. The patient is normally in a supine or semi-recumbent position during the test, which is further divided into two separate stages: the filling stage and the bladder emptying stage.

During cystometry, sensation in the bladder, the compliance of the bladder walls, and bladder capacity are determined.

### 4.2. Voiding Cystometry

Voiding cystometry is the final part of cystometry. The examination is based on passing urine freely with inserted catheters in the bladder and rectum. Voiding cystometry allows for the differentiation between reduced detrusor contractility and bladder outlet obstruction (BOO). The pressure inside the bladder is the sum of the intra-abdominal pressure (Pabd) and the pressure that is produced by the bladder detrusor (Pdet). The pressure generated by the detrusor muscle of the bladder is calculated while using the following formula:

Pdet = Pves-Pabd5

## 5. Profilometry

In urethral profilometry, resting and stress urethral pressures are measured, alongside the functional length of the urethra. Rest pressures that are below 20 cm H_2_O indicate damage to the urethral sphincter mechanism. Additionally, urethral instability can be found, allowing us to understand involuntary urinary incontinence, which is unrelated to effort and urge, with normal bladder function in the urine collection and urine output phases.

In summary, one should remember to standardize the terminology for female pelvic floor dysfunction, as pointed out by the International Urogynecological Association (IUGA)/International Continence Society (ICS) Joint Report. It is helpful in everyday clinical practice. This recommendation gives the definition of: ideal conditions for free (or spontaneous—no catheter) uroflowmetry, Urine flow, flow rate, voided volume (mL), maximum (urine) flowrate (MUFR, mL/s)—Q_max_, maximum (urine) flow rate (MUFR, mL/s)—Q_max_, Flow time (s), average (urine) flow rate (AUFR, mL/s)—Q_ave_, voiding time (s), time to maximum flow (s), and interpretation of the normality of free uroflowmetry [19]. In our description of the uroflowmetry study, we used the recommended definitions.

In the examination, only those patients were included, in whom, before treatment, no urinary incontinence was determined, and who had no recurrence after oncological treatment. Women in the control group and those in the two study groups were treated for three, six, nine, and 12 months after the completion of treatment or occurrence of symptoms.

Patients were meant to complete a follow-up in the 3rd, 6th, 9th, and 12th months after the operation. In the case of the occurrence of symptoms, urogynecological examinations were also conducted to verify the diagnosis. During the follow-up, gynecological examination was conducted with particular attention being paid to urogynecological aspects, as well as the UDI-6 survey and the quality of life questionnaire II-Q7. Subsequently, the results obtained from the surveys were analyzed and interpreted. In the case of an occurrence of disorders in the urinary system, urogynecological examination was conducted in order to clarify the cause of these symptoms.

All of the surgeries were done in the Department of Gynecology and Obstetrics with Gynecologic Oncology, with complementary chemotherapy in the Department of Clinical Oncology, whereas radiation therapy was done in the Department of Radiation therapy, bladder function tests and questionnaires on the quality of life were assessed at the Urodynamic Clinic of at the Ludwik Rydygier Memorial Specialized Hospital in Kraków. In the examined women, a medical interview was completed, and gynecological and urodynamic tests were done, excluding the inflammatory process. Every woman from groups A, B, and C was asked about completing the following questionnaires: the SWLS, in which the psychological state of the patients was reviewed; II-Q7; UDI-6. The results of urinary tract examinations were interpreted in four possible diagnoses:

1. No symptoms connected with the urinary system.
2. Stress urinary incontinence (SUI).
3. Overactive bladder (OB).
4. Mixed Urinary Incontinence (MUI).

The SWL survey on the quality of life was assessed in three possible situations:

1. State unchanged.
2. Decrease in quality of life.
3. Increase in quality of life.

For the analysis of data, the STATISTICA 13.0 (StatSoft, Cracow, Poland) packet was used. The Chi-square test was used (Chi2) to test the relationship between qualitative characteristics. Statistically significant results were considered to be those that were below the test probability level (*p*) of 0.05 (*p* < 0.05). The results of the test were compared between groups: group A vs. C; group B vs. C; group A vs. B; and, groups A and B vs. C. In the last stage, the Pearson correlation analysis between the questionnaires-II-Q7 and UDI-6-determined the type of urinary incontinence with the results from the urogynecological examination (*p* < 0.05). Additionally, based on Cronbach’s alpha coefficient, the reliability and consistency of the questionnaires (II-Q7 and UDI-6) were determined, while assuming the value of the coefficient being greater than or equal to 0.7 as satisfactory.

## 6. Results

Firstly, the frequency at which the following forms of urinary incontinence appeared was analyzed: stress urinary incontinence; overactive bladder; mixed urinary incontinence, this was in patients after three, six, nine, and 12 months from the completion of combination therapy due to endometrial or ovarian cancer compared to patients, in whom endometrial cancer did not require the use of brachytherapy after the removal of the uterus. The smallest influence on the functioning of the urinary system can be observed in the control group, where the number of patients not reporting a disorder in the urinary system ranges from 72–76 patients. In the group of patients with endometrial cancer, it can be observed that, after six months from the completion of treatment, the number of patients without symptoms decreased compared to the time period for three months after the completion of treatment 24 vs. 35); however, with longer observation, a small increase in the number of patients without a disorder of the urinary system was observed. The most common form of urinary incontinence in this group was mixed urinary incontinence, next it was stress urinary incontinence and then the overactive bladder. In turn, in the group of patients after the completion of treatment of ovarian cancer, a lack of negative effects on the urinary system was noted in 32–42% of the women (depending on the time period when the examination was carried out). The smallest number of patients in the discussed group, in whom urogynecological examination excluded urinary incontinence six months after the completion of therapy. Afterward, in the 9th and 12th month after the completion of treatment, the number of patients without a disorder gradually increased (independently from the treatment period, statistically significant differences were observed during the comparison of traits between the groups: A vs. C; B vs. C; A + B vs. C; and, A vs. B (Table 1; *p* < 0.05).

Next, based on the questionnaire SWLS, the quality of life belonging to the three analyzed groups as part of this work was assessed. The decrease in the quality of life in the group of patients, which completed combination therapy due to ovarian cancer, was declared by 53% of women after three months, and this value only fluctuated slightly throughout the treatment (54%—six months, 53%—nine months, 50%—12 months). The percentage of patients, in whom treatment did not affect the quality of life at all, was relatively high (approximately 40% of those treated). In turn, in the group of patients after completed treatment as a result of endometrial cancer, the decrease in quality of life was determined for 59–62% of patients, depending on the month after the completion of treatment when the SWLS questionnaire was completed. Additionally, the number of patients who determined that the treatment did not affect, in any way, their quality of life, was lower than the group of patients with ovarian cancer and was 36% at the beginning of observation and, after six months, it was 32% for 9 months after the completion of treatment and 35% after a year had passed.

The smallest percentage of patients, which noted a decrease in their quality of life could be observed in the group of women who did not undergo adjuvant treatment (4–9%). Simultaneously, in this group, the highest case of improvements in the quality of life after treatment is determined. Statistical analysis indicated the occurrence of statistical significance, at every point of time between groups: A vs. C; B vs. C; and, A + B vs. C (*p* < 0.05; Table 2).

In the next stage, the usefulness of using questionnaires II-Q7 and UDI-6 in certain types of urinary incontinence was assessed (Table 3 and Table 4).

As a part of this goal, correlation analysis between the recognized type of urinary incontinence as a survey result and the results of urogynecological examination. It was observed that, for the questionnaire II-Q7, the correlation coefficient was: for stress urinary incontinence was r = +0.69 (*p* < 0.05); for neurogenic bladder r = +0.30 (*p* > 0.05); and for mixed urinary incontinence r = +0.89 (*p* < 0.05). In turn, for the UDI-6 questionnaire the following interdependence was determined: stress urinary incontinence r = +0.89 (*p* < 0.05); for neurogenic bladder r= +0.61 (*p* > 0.05); and, for mixed urinary incontinence r = +0.48 (*p* < 0.05).

The last stage of analysis was connected with the assessment of the reliability of the questionnaires II-Q7 and UDI-6, where the value of Cronbach’s alpha coefficient was, respectively, 0.94 and 0.89; therefore, the criterion for the value to be equal to or higher than 0.7 was fulfilled.

## 7. Discussion

Cancer is a traumatic experience for women. It forces a lifestyle change and limits the fulfillment of life roles [17,18]. Patients have to face situations that burden the psyche and, sometimes, they can be beyond their ability to cope with stress [20,21]. Women during and after the completion of oncological treatment tend to worry about a loss in their attractiveness, a worsening in their relationship with their partner and surroundings [22]. Furthermore, they may experience anxiety and restlessness connected with cancer, side effects of treatment, pain, and above all the loss of life. Cancer requires modifications in life plans, which can also impact the quality and comfort of life in a negative way [23,24]. The appearance of a disorder in regards to the urinary system in the form of urinary incontinence is one of the most commonly found side effects of combination therapy due to ovarian or endometrial cancer [10,11]. Therefore, it is also fully justified to assess combination therapy depending on its type, which is in turn determined by the location of the neoplastic lesion for urinary incontinence and the assessment of the quality of life after oncological treatment [12,13]. The problem of disruptions in the functionality of the urinary system in oncological patients is usually underestimated, as it is of secondary importance in the context of the health situation that is associated with a malignant tumor [10,11]. Nonetheless, the problem should not be belittled, as is indicated by the observations of Strauchon et al. [14] and Ramaseshan et al. [16]. On the other hand, the analysis conducted by Bretschneider et al. did not indicate that the frequency of occurrence of disruptions in urination was dependent on the type of cancer. However, it was noted that pain in the bladder was statistically significantly more frequently observed in women with gynecological tumors before 50 years of age, whereas, in women over the age of 50, the urge to urinate was more frequently observed [25]. In turn, White et al. determined that urinary incontinence problems occurred more often in patients with endometrial cancer in comparison to healthy women (odds ratio [OR]: 1.31; *p* < 0.05) [26].

In accordance with the applicable recommendations regarding conducting treatment in the case of endometrial and ovarian cancer, the radical removal of the uterus with appendages should always be done. Next, depending on the stage of neoplastic changes, in the case of endometrial cancer, patients undergo adjuvant therapy-brachytherapy, and, in turn, post-operative patients of ovarian cancer are directed to chemotherapy [1,7,8,9]. As part of this study, we assessed the quality of life as well as the frequency of appearance of urinary incontinence and its breakdown into different forms in the first year after the completion of combination therapy in patients with recognized endometrial and ovarian cancer. Furthermore, the attempt to determine the utility of using the scales IILQ-7 and UDI-6 in the diagnosis and differentiation of urinary incontinence. Another strength of the study was the fact that one gynecologist specializing in urogynecology and oncological gynecology as well as one clinical psychologist undertook the assessment of the health condition for the whole analysis time period. Moreover, patients in whom before treatment began there was a urinary incontinence problem were not included in the study.

By analyzing the results of the SWL scale, it was observed that the largest percentage of oncological patients, which declared a decrease in their quality of life after treatment, was the group of women with recognized endometrial cancer, in whom surgery and brachytherapy were used. Additionally, in this group, the lowest percentage of patients as compared to the other two groups was noted to have had a neutral influence on their quality of life. Likewise, in the group of patients after the completion of ovarian cancer treatment, the number of women assessing their quality of life as worse in comparison to the time period before the treatment was statistically higher in comparison to the group of women with endometrial cancer, whose treatment was completed by carrying out a radical removal of the uterus with appendages. Interestingly, there were no statistically significant differences in the quality of life (*p* > 0.05) between the two groups in whom combined therapy was used. In endometrial and ovarian cancers, the scope of the operation to remove the uterus is usually classified as Piver II, and the radicality of the operation of endometrial cancer mainly depends on the removal of the vaginal cuff and lymph nodes. Whereas in ovarian cancer, the extent of the procedure is connected with lymph node resection as well as other organs mainly from the abdominal cavity [27,28]. In the studies of Oui et al. [29] and Oplawski et al. [30], this sort of uterus removal should not have a significant influence on urinary incontinence [29,30]. Complications that are related to the statics of the pelvic floor leading to urinary system disorders are believed to be significantly less likely after the Piver II procedure than the Piver III procedure, which is used in cases of cervical cancer [29,30]. The research that was conducted by Donovan et al. [31] is also interesting; they determined that significantly more often urinary incontinence problems occurred in the group of patients that underwent oncological treatment (23%—radical hysterectomy; 70%—simple hysterectomy; 29%—chemotherapy; 27%—tele- and brachy radiotherapy). Most often, they observe: nocturia (OR: 2.7, *p* < 0.001); urge to urinate (OR: 3.5, *p* < 0.0005); uncontrolled leakage of urine at least once a day (OR: 3.7, *p* < 0.006); and, pain in a full bladder (OR: 3.1, *p* < 0.005). In turn, symptoms of urinary incontinence (OR: 2.2, *p* < 0.01), SUI (OR: 2.1, *p* < 0.02), MUI (OR: 2.9, *p* < 0.0008), and involuntary urination (OR: 5.7, *p* < 0.0001) were not significant in terms of micturition disorders. Bladder capacity disturbances were most often a consequence of radical hysterectomy and radiotherapy, while the symptoms of urinary incontinence were correlated with body mass index and with radiation therapy. Radical hysterectomy is associated with a high risk of complications from the urinary system, due to its extent and the associated risk of damaging the vegetative innervation of the pelvic organs. Symptoms of urinary incontinence were significantly more frequent among patients who underwent radical hysterectomy as compared to patients who underwent simple hysterectomy (57% vs. 32%; *p* < 0.01) [31].

The method that allows for the limiting of the frequency of complication rates is radical hysterectomy sparing the pelvic vegetative innervation (NSRH), which is recommended in cases of stage IB1/IB2 cervical cancer, according to the classification of the International Federation of Gynecology and Obstetrics (FIGO) from 2018 in patients, in which type C1 hysterectomy of the Querleu–Morrow classification is planned [32]. The results of the meta-analysis that was conducted by Xue et al. suggest that NSRH lowers the risk of developing bladder dysfunctions in comparison to the radical hysterectomy does (relative risk: 0.17) [33]. Because, after NSHR, the catheterization time is shorter than in the case of radical hysterectomy, the risk of urinary incontinence problems occurring is lower. Radiotherapy conducted after radical hysterectomy significantly disturbs the bladder function parameters. In comparison to patients, in which only the operation was conducted, in patients who underwent adjuvant radiotherapy, a significantly lower maximum bladder volume and compliance was determined, as well as lower urine volume that caused a strong urge to urinate, not only 10 days after the operation, but also six months after it [34,35].

Interesting insights on cytostatic therapy (carboplatin/paclitaxel) in patients with ovarian and endometrial cancer were described by Strauchon et al., who completed the assessment using the questionnaires UDI-6 and IILQ-7 before beginning treatment, in the fifth cycle of treatment as well as between the 6th and 12th week after the completion of therapy. They assume that chemotherapy could in some negatively impact the functioning of the urinary system; however, more studies for to determining whether symptoms are transient or permanent need to be done [14].

However, the effect of the surgical treatment of uterine fibroids on the symptoms of LUTD is different. Dancz et al. obtained a significant postoperative improvement in terms of the urgency to urinate, incomplete emptying of the bladder, NMPN, the frequency of voiding, and bladder pain [36].

The currently used techniques of “nerve-sparing” also positively reduce the risk of damage being done to the nervous system of patients undergoing surgery, regardless of whether traditional or minimally invasive operations utilizing this technique are compared [16,24]. In the study that was carried out by Laterza et al., they indicate that a disorder in the urinary system after a radical operation has a neurogenic etiology, including the important role of urethral sphincter compression in postoperative urinary incontinence. What is especially important is that using “nerve-sparing” techniques does not associate with a decrease in survival time [37]. It is suggested that the appearance of a urinary system disorder in the case of endometrial cancer is closely related to brachytherapy [38,39], which is consistent with our observations. Based on the results of urodynamic tests, we observed that in the group that underwent brachytherapy, problems with urinary incontinence are twice as likely when compared to cases where chemotherapy is used as the adjuvant treatment. This is also confirmed by the analysis of Choo et al. that was carried out on a group of men with prostate cancer, who were being treated using irradiation therapy, indicating urologic problems as the main element decreasing their quality of life [40]. Data regarding the influence of chemotherapy on urinary system disorders are fragmented. Additionally, Murata et al. indicate that chemotherapy, which is used in hematological cancers, can negatively impact the urinary system [41]. Our observations carried out for one year after the completion of combination therapy suggest that, in most cases, changes in the functioning of the urinary system in the form of urinary incontinence are permanent changes. This is confirmed by the fact that, with more time passing from the completion of combination therapy, the number of patients in whom the urogynecological examination did not a problem, the problem with urinary retention increased slightly. Likewise, the analysis that was conducted by Ziętek-Strobl et al. also indicates the continuation of disorders in the urinary system after oncological treatment, which included surgery and adjuvant treatment, due to endometrial, cervical, vulval, and ovarian cancer, after six months of observation, the number of patients who reported a problem with urinary incontinence increased from 69 to 78. These authors also indicate that the risk of the problem in question occurring is not connected with the type of gynecological cancer, but rather depends on the extent of the surgery [42]. Because of this, it is indicated that reduced risk surgery should be considered in order to reduce the risk of damaging urogenital nerves and blood vessels [43,44,45,46]. Mixed urinary incontinence was the most commonly occurring form of urinary incontinence in the group of ovarian cancer patients and, in the case of endometrial cancer, the dominating forms were mixed and stress urinary incontinence. Urogynecological examination was supplemented with questionnaires regarding the quality of life of patients with urinary incontinence-II-Q7 as well as UDI-6 and, by this or based on the results that were obtained after their completion, it was determined whether it is possible to determine the type of urinary incontinence using them. It can be determined that the questionnaire II-Q7 is the most useful in the diagnosis of mixed urinary incontinence (r = +0.89) as well as stress urinary incontinence (r = +0.69). Whereas, the questionnaire UDI-6 seems to be the most useful in recognizing stress urinary incontinence (r = +0.89) as well as the neurogenic bladder (r = +0.61). The reliability of the obtained results from the survey research was determined at 0.94 (II-Q7) and 0.89 (UDI-6) by Cronbach’s alpha coefficient value, which is also consistent with the observations of Faruqui et al. [47]. In turn, Utomo et al. determined that Cronbach’s alpha coefficient value for II-Q7 was 0.87 and 0.49 for UDI-6 [48], which is lower than in our study and in the analysis that was carried out by Faruqui et al. [47], where the value of the discussed index for the scales of both scales was above 0.7. The discrepancy between the results that were obtained by us and the results of other researchers [46,47] can be resultant from differing group sizes or the fact that, in the cited works, the patients were not tested oncologically. These results indicate that a universal questionnaire does not exist on which it is possible to qualify someone to one of the three urinary incontinence groups. It is recommended to continue further studies in the field of urogynecology after oncological treatment and a comparison of the results of the questionnaires: SWL; UDI-6: II-Q7 between the groups of patients with endometrial and ovarian cancer after combination therapy, the group of patients in whom treatment was limited to just surgery and the group of women with no oncological burden, having symptoms of urinary incontinence.

## 8. Conclusions

Urinary incontinence is an often appearing side effect of combination therapy of endometrial and ovarian cancer. The risk of a urinary incontinence problem appearing in female patients increases alongside an increase in the radicality of the nature of treatment, as well as the use as adjuvant therapy-brachytherapy in comparison to chemotherapy. It also seems that the changes in the urinary system have a fixed character, which is indicated by the fact that there is a relatively low decrease in the number of female patients who did not report urinary tract complaints after 12 months of observations in comparison to the first observation in the third month after the conclusion of treatment. The II-Q7 and UDI-6 questionnaires can serve as supplementary tools in the diagnosis of urinary incontinence.

## Figures and Tables

**Table 1:** Comparison of urogynecological examination in the study groups.

Time from the Completion of Treatment (Months)	Result of Treatment	N			*p*-Value
		Group C	Group A	Group B	
3	No changes (NC)	74	14	35	0.0191 *
	Stress urinary incontinence (SUI)	5	1	10	0.0085 **
	Overactive Bladder (OAB)	3	20	20	0.0288 ***
	Mixed urinary incontinence (MUI)	5	3	27	0.0299 ****
6	No changes (NC)	72	12	24	0.0161 *
	Stress urinary incontinence (SUI)	4	1	21	0.0082 **
	Overactive Bladder (OAB)	5	21	16	0.0018 ***
	Mixed urinary incontinence (MUI)	6	4	26	0.0236 ****
9	No changes (NC)	72	15	25	0.0129 *
	Stress urinary incontinence (SUI)	4	1	20	0.0077 **
	Overactive Bladder (OAB)	5	18	15	0.0287 ***
	Mixed urinary incontinence (MUI)	6	4	27	0.0181 ****
12	No changes (NC)	76	16	24	0.0128 *
	Stress urinary incontinence (SUI)	5	1	16	0.0072 **
	Overactive Bladder (OAB)	2	17	14	0.0286 ***
	Mixed urinary incontinence (MUI)	4	4	33	0.0181 ****

* C vs. A, ** C vs. B, *** A vs. B, **** C vs. (A + B)-statistically significant differences (*p* < 0.05).

**Table 2:** Results of the SWLS survey assessing the quality of life.

Time from the Completion of Treatment (Months)	Result of Treatment	N			*p*-Value
		Group C	Group A	Group B	
3	No changes	36	15	31	0.0014 * 0.0055 ** 0.8642 *** 0.0002 ****
	Better	45	3	3	
	Worse	6	20	58	
6	No changes	38	16	35	0.0013 * 0.0055 ** 0.8282 *** 0.0002 ****
	Better	41	1	4	
	Worse	8	21	53	
9	No changes	37	18	36	0.0014 * 0.0057 ** 0.8501 *** 0.0002 ****
	Better	42	2	2	
	Worse	8	18	54	
12	No changes	42	16	34	0.0015 * 0.0054 ** 0.8642 *** 0.0019 ****
	Better	38	3	6	
	Worse	7	19	52	

* Cvs. A, ** C vs. B, *** A vs. B, **** C vs. (A + B)–statistically significant differences (*p* < 0.05).

**Table 3:** Results of the Urogenital Distress Inventory (UDI-6) survey.

Time from the Completion of Treatment (Months)	Answer Variant	Do You Urinate Frequently?			Do You Find There is Leakage of Urine Associated with the Feeling of Pressure on the Bladder?			Do You Find There is Leakage of Urine Due to Physical Activity, Coughing, or Sneezing?			Do You Find There is Leakage of Urine in Small Amounts (Droplets)?			Do You Have Problems with Emptying Your Bladder?			Do You Have Pain in the Lower Abdomen or around the Vulva?		
		C	A	B	C	A	B	C	A	B	C	A	B	C	A	B	C	A	B
3	No	71	15	35	75	33	40	75	33	46	77	33	48	80	35	79	77	33	75
	Not at all	0	0	0	0	0	0	0	0	0	0	0	0	0	0	0	0	0	0
	Some/A little	8	3	14	3	3	15	3	1	10	1	1	25	6	2	10	6	3	10
	Medium	2	12	16	4	1	20	5	2	19	3	1	12	1	1	3	4	2	5
	Very/A lot	6	8	27	5	1	17	4	2	17	6	3	11	0	0	0	0	0	2
6	No	68	13	33	73	34	44	74	33	46	76	33	50	81	36	80	80	35	82
	Not at all	0	0	0	0	0	0	0	0	0	0	0	0	0	0	0	0	0	0
	Some/A little	6	7	15	4	2	15	3	1	11	1	0	27	6	1	10	5	2	8
	Medium	3	10	17	2	1	18	2	1	18	6	2	12	0	1	2	2	1	2
	Very/A lot	10	8	26	8	1	16	8	3	17	4	3	9	0	0	0	0	0	0
9	No	68	16	35	73	33	46	73	32	48	76	33	50	82	36	82	82	35	85
	Not at all	0	0	0	0	0	0	0	0	0	0	0	0	0	0	0	0	0	0
	Some/A little	6	8	15	3	3	14	2	2	13	1	1	28	5	2	9	3	3	5
	Medium	3	10	12	3	1	19	3	1	18	5	1	11	0	0	1	2	0	2
	Very/A lot	10	4	25	8	1	14	9	3	17	5	3	9	0	0	0	0	0	0
12	No	75	17	40	77	34	46	77	34	48	78	33	51	84	36	84	84	36	85
	Not at all	0	0	0	0	0	0	0	0	0	0	0	0	0	0	0	0	0	0
	Some/A little	5	8	15	1	1	16	1	1	15	0	1	28	3	2	8	3	2	7
	Medium	2	9	27	2	1	17	3	2	17	5	2	10	0	0	0	0	0	0
	Very/A lot	5	4	10	7	1	14	6	1	16	4	2	9	0	0	0	0	0	0

**Table 4:** Results of II-Q7 survey.

Time from the Completion of Treatment (Months)	Answer Variant	Does Leakage of Urine and/or Lowering/Prolapse of the Vaginal Walls/Reproductive Organ Affect You in the Following Categories:																				
		Ability to Do Household Activities			Physical Recreation Such as Walking, Swimming			Entertainment, Such as Going to the Cinema or a Concert			Possibility to Travel by Car or Bus for Longer than 30 min from the House			Participation in Social Activities Outside the Home			Mental/Emotional Health			Feeling Frustrated		
		C	A	B	C	A	B	C	A	B	C	A	B	C	A	B	C	A	B	C	A	B
3	No	80	18	55	72	20	40	81	23	50	75	28	55	71	25	61	79	15	34	72	11	34
	Not at all	0	0	0	0	0	0	0	0	0	0	0	0	0	0	0	0	0	0	0	0	0
	Some/A little	5	12	20	13	12	12	5	13	30	10	7	25	15	12	20	5	5	31	12	9	50
	Medium	2	5	11	2	5	23	0	2	10	2	3	11	1	1	9	3	10	20	3	10	7
	Very/A lot	0	3	6	0	1	17	0	0	2	0	0	1	0	0	2	0	8	7	0	8	0
6	No	80	21	56	72	21	42	81	25	55	76	29	60	74	27	65	79	16	39	76	13	39
	Not at all	0	0	0	0	0	0	0	0	0	0	0	0	0	0	0	0	0	0	0	0	0
	Some/A little	6	12	19	14	12	12	5	12	30	11	7	26	12	10	25	6	8	31	12	8	51
	Medium	1	5	11	1	5	23	0	1	7	0	2	6	1	1	2	2	5	19	0	8	2
	Very/A lot	0	0	6	0	0	15	0	0	0	0	0	0	0	0	0	0	9	3	0	9	0
9	No	81	25	60	74	23	48	83	25	59	79	29	61	76	27	69	80	18	38	79	14	40
	Not at all	0	0	0	0	0	0	0	0	0	0	0	0	0	0	0	0	0	0	0	0	0
	Some/A little	6	11	20	12	12	12	4	13	31	9	8	27	10	11	21	6	10	32	8	9	50
	Medium	0	2	10	1	3	23	0	0	2	0	1	4	1	0	2	1	5	18	0	9	1
	Very/A lot	0	0	2	0	0	9	0	0	0	0	0	0	0	0	0	0	5	4	0	6	0
12	No	82	26	63	74	22	50	86	27	59	85	29	69	80	29	74	80	16	40	81	19	39
	Not at all	0	0	0	0	0	0	0	0	0	0	0	0	0	0	0	0	0	0	0	0	0
	Some/A little	5	11	21	13	11	23	1	11	33	2	9	23	7	9	18	7	10	39	6	10	53
	Medium	0	1	7	0	5	12	0	0	0	0	0	0	0	0	0	0	6	13	0	6	0
	Very/A lot	0	0	1	0	1	7	0	0	0	0	0	0	0	0	0	0	6	0	0	3	0

## Data Availability

The data used to support the findings of this study is included in the article. The data will not be shared due to the fact the third-party rights and commercial confidentiality.
